# The role of early-onset-sepsis in the neurodevelopment of very low birth weight infants

**DOI:** 10.1186/s12887-021-02738-5

**Published:** 2021-06-25

**Authors:** Tjark Ortgies, Michael Rullmann, Dorothée Ziegelhöfer, Annett Bläser, Ulrich H. Thome

**Affiliations:** 1 Division of Neonatology, University Children’s Hospital Leipzig, Liebigstraße 20, 04103 Leipzig, Germany; 2 Department of Nuclear Medicine, University Hospital Leipzig, Liebigstraße 18, 04103 Leipzig, Germany

**Keywords:** Early-onset-Sepsis, Neurodevelopment, Preterm, VLBW

## Abstract

**Aims:**

The study investigated a putative association between early-onset-sepsis (EOS) and poor neurodevelopmental outcomes at 2 years corrected age in very low birth weight infants.

**Methods:**

This was a single-center cohort study on infants weighing less than 1500 g with a gestational age below 35 weeks at birth born between 2008 and 2011. Neurodevelopmental outcomes were assessed at follow-up with the Bayley Scales of Infant Development-II. EOS was defined as either culture-proven EOS or clinical EOS using blood culture, CrP levels, and clinical symptoms and treatment. Neurodevelopmental impairment (NDI) was defined as one or more of the following: Mental Developmental Index (MDI) and/or Psychomotor Developmental Index (PDI) scores lower than 70; presence of cerebral palsy.

**Results:**

Of 405 eligible newborns in the study period 166 were included. Two had culture-proven and 29 clinical EOS. Median MDI scores in patients with EOS were 96 (IQR: 86–106) and in the control group 94 (84–106, *p* = 0.77). PDI scores in patients with EOS were 96 (86–106) and in the control group 99,5 (92–103, *p* = 0.03). Of infected patients 7/31 (24%) showed NDI as defined, whereas only 11/135 (8%) showed NDI in the control group (OR 3.3, *p* = 0.03). Multiple regression analyses identified chorioamnionitis and poor CRIB-Scores as individual risk factors for MDI or PDI values < 70.

**Conclusion:**

In our study, EOS among VLBW-infants significantly impaired the neurodevelopment at 2 years corrected age. As shown in previous reports infection continues to be a problem and strategies for a reduction need further improvement.

## Introduction

Very low birth weight infants (VLBW, birth weight < 1500 g) are more susceptible to brain injury than term infants. This may lead to adverse long-term neurodevelopmental outcomes [1, 2]. While survival rates of VLBW-infants have increased in recent years, the rates of long-term developmental impairments have not decreased [3, 4].

In their review article, Saigal et al. [2] pointed out that about one quarter of surviving preterm infants have substantial neurological morbidity. Common impairments affecting the central nervous system (CNS) are intellectual disability, cerebral palsy, and sensory impairments [1]. Several mechanisms have been proposed as to how systemic inflammation and subsequently elevated cytokine levels in mother and child in the perinatal period may damage brain parenchyma [5–7].

Other well established risk factors for adverse long-term neurological outcomes are low gestational age, low birth weight, male gender, bronchopulmonary dysplasia (BPD), intraventricular hemorrhage (IVH), necrotizing enterocolitis (NEC), periventricular leukomalacia (PVL), and retinopathy of prematurity (ROP) [2, 8, 9].

The aim of our study was to assess the effects of early-onset-sepsis (EOS) on VLBW infants’ neurological development. EOS is usually defined as onset of an infection within the first 72 h of life in hospitalized infants with a proof of pathogen by blood culture [9–11]. Numbers regarding the incidence of culture-proven EOS in VLBW infants range from 1 to 28 per 1000 live births depending on gestational age, birth weight, ethnicity and intrauterine infection among many other factors [9, 10, 12].

However, clear sepsis symptoms can occur even though attempts to culture the causative organism may remain unsuccessful [4, 13]⁠. This situation has been defined as clinical sepsis in similar research [6, 8, 10, 14–16]. Recognizing clinical sepsis can be enhanced by long established blood biomarkers such as C-reactive protein (CrP) and interleukins [15, 17, 18]⁠. The definition of EOS as an infection starting within the first 72 h of life reflects the proposed pathophysiology, as pathogens are considered to be transmitted vertically from mother to child in the pre- and perinatal period [4, 9, 10]⁠. Pathogens causing EOS are most commonly Group-B-*Streptococcus* (GBS) and, increasingly, *Escherichia coli* and several less common bacterial pathogens, but they may also include candida or viral pathogens [4, 9, 10, 12, 19].

Few reports have been published regarding a possible negative effect of infection on neurodevelopmental outcomes in premature infants. These reports were predominantly based on infants born in the 1990s and included both entities of neonatal infection (EOS and LOS) [6, 8, 20, 21]. The most recent study evaluated infants with either a positive blood culture or prolonged antibiotic treatment, which, however, was solely based on clinical symptoms [11]⁠. According to the above mentioned mechanisms causing damage to brain parenchyma, the immaturity of the CNS is believed to be a substantial factor [5, 7]⁠. Therefore, in our study we focused on patients with EOS and clinical-EOS using a more rigorous clinical EOS definition, which included mandatory laboratory abnormalities. We hypothesized that clinical as well as culture-proven EOS might be associated with adverse neurodevelopmental outcomes.

## Methods

### Patients and data acquisition

The cohort of our retrospective study included all inborn VLBW infants with a gestational age of less than 35 weeks cared for in our tertiary care neonatal intensive care unit (NICU) in the years 2008 through 2011. Data was retrieved from the hospital records of these patients and their mothers from during their stay in our hospital. Patients with congenital defects and syndromes were excluded from our cohort. Equally patients, who were exposed to maternal drug abuse during pregnancy and received postnatal opiate substitution, and those with incomplete hospital records were excluded, as were patients who died*.*

Only patients who returned for a follow-up visit around the corrected age of 24 months for the neurodevelopmental assessment were included in the final analysis. Basic demographic and clinical data of patients with follow-up were compared with data of patients lost to follow-up.

### Definitions

Culture-proven EOS was defined as a positive result of one or more bacterial or fungal blood cultures obtained from patients and antimicrobial treatment for at least 5 days. As blood culture has a low sensitivity, especially when limited blood volumes are available, we used a definition of clinical EOS as done in previous similar studies [6, 8, 13, 16, 22]⁠. We defined clinical EOS using established laboratory parameters and persistent clinical presentation [15, 17, 18]:

1. either patients show a CrP ≥ 10 mg/L in the first 72 h of life and receive antimicrobial treatment for at least 5 days
2. or patients with a CrP ≥ 5 mg/L have undergone a course of antimicrobial therapy of more than 5 days and present with three or more persistent clinical symptoms as defined below [23]⁠

The following clinical symptoms have been identified in prior research as suggesting an infection and as such have been implemented in German national guidelines [4, 13, 23–27]⁠. These were, in no particular order:

- Temperature instability such as fever > 38.0 °C, hypothermia < 36,5 °C, frequent adjustment of the incubator.
- Prolonged capillary refill > 2 sec.
- Apnea defined as new or more frequent episodes > 20s.
- Tachycardia of > 200 beats/minute.
- Poor feeding (increased difficulty in tolerating enteral feeding, i.e. repeated vomiting).
- Hypotension presenting with a mean arterial pressure in mmHg less than gestational age in weeks, or a significant decrease in the patients blood pressure leading to a use of inotropic drugs or pallor.
- Increased oxygen demand with any increase in the amount of oxygen needed to obtain an oxygen saturation of 88–92% persisting > 60 min.
- Acidosis presenting with a base excess < − 10 mEq/l.

In addition we looked at an increase of interleukin-6 laboratory (IL-6, cut-off ≥50 pg/ml) which, in combination with the above mentioned CrP, has been established over decades as a sensitive marker in the diagnosis of EOS [15, 17, 18]⁠. Given that there is a time lag of 12–24 h as well as a poor positive predictive accuracy in a single value, increasing serial CrP values in the same recorded infection period, also past the first 72 h of life, were taken into the consideration as well [15]⁠. If there were multiple CrP values at hand, the highest was used in the analyses. Abnormal white blood cell counts (WBC) were recorded using percentiles of VLBW-Infants on the third day of life as published by Obladen et al. [28], with leukocytosis being > 24,5 Gpt/L (90th percentile) and leukopenia being < 4,8 Gpt/L (10th percentile). Thrombocytopenia was recorded as a platelet count < 150,000 Gpt/L and severe thrombocytopenia as a platelet count < 50,000 Gpt/L [29]⁠.

The common denominator of the EOS classification was the incidence of systemic infection in the first 72 h of life, using established paraclinical parameters and known clinical symptoms evaluated by experienced neonatologists, and leading to antimicrobial therapy for at least 5 days.

The group without EOS consisted of all inborn VLBW infants born during the same time interval but not meeting the criteria of infection within the first 72 h of life. Patients suffering from infection past 72 h of life were considered to have LOS [9]⁠.

NEC was diagnosed according to the criteria of Bell et al. [30], with clinical and radiological findings meeting the definition of stage II or higher. As in previous studies, patients with NEC (stage II or higher) were considered to be septic as well, as there is a strong association of NEC with infection [6, 8, 31].

ROP was routinely screened during hospitalization according to the international classification [32]. ROP was considered an adverse outcome, if stage 3 or higher was attained [8]. The incidence and severity of IVH were routinely assessed by cranial ultrasounds after birth, within 2 weeks after birth and prior to discharge, classified according to Papile et al. [33]. Brain injury was interpreted as IVH grade 3 or higher [3, 8, 34]. PVL was assessed during these exams as well, according to the classification of Vries et al. [35], and it was considered as an adverse outcome, if persistent white matter injury (stage 3 or higher) was diagnosed prior to discharge. BPD was characterized as oxygen being supplemented or positive-pressure support being given at 36 weeks postmenstrual age, meeting the criteria of moderate to severe BPD according to the National Institute of Child Health and Development consensus definition [36, 37]. Chorioamnionitis was recorded when a clinical diagnosis according to the hospitals standard operating procedure (maternal fever, persistent elevated temperature, fetal tachycardia > 160 min longer than 10 min, maternal leukocytosis, purulent discharge) was confirmed histopathologically or through elevated IL-6 levels in amniocentesis [38]⁠.

Z-scores for weight, length, and head circumference were calculated at birth, based on German percentiles by Voigt et al. [39]. The Clinical Risk Index for Babies (CRIB) score, including scores for gestational age, birthweight, maximum and minimum fraction of inspired oxygen and maximum base excess during the first 12 h of life, was also determined in infants meeting the gestational age criterion of birth below 32 weeks. The criterion of congenital malformation within the CRIB score was irrelevant in our study due to the exclusion criteria [40].

To evaluate the influence of socioeconomic status, parents were subdivided into 4 groups according to information given by parents at admission to hospital as follows: one or more higher educated caregivers with any academic degree (category 4), one or more caregivers having successfully completed a professional training (category 3), caregivers with a high school diploma (category 2) or those without any degree (category 1) [8, 41].

### Outcome assessment

All surviving infants were invited to a neurodevelopmental follow-up at 2 years ± 3 months corrected age using the Bayley Scales of Infant Development (BSID) II in their German translation [42]. The assessment was carried out by experienced neonatologists (AB) as part of routine follow-up examinations in the hospitals neonatology division.

In addition, motor function was assessed according to the modified Gross Motor Function Classification System (GMFCS) to determine cerebral palsy [43].

Neurodevelopmental impairment (NDI) was defined as one or more of the following: Mental Developmental Index (MDI) and/or Psychomotor Developmental Index (PDI) scores lower than 70; presence of cerebral palsy. Furthermore, MDI or PDI scores < 85 in the absence of cerebral palsy were considered as a secondary outcome [6, 8].

### Statistical analyses

Statistical significance for unadjusted comparisons was determined by appropriate tests, including ANOVA, Kruskal-Wallis, χ^2^ with and without Yates’ correction for continuity, Mann-Whitney U, student’s t-test including the Welch’s correction, and Fisher’s exact tests. As this was an exploratory analysis, a *p* value of < 0.05 was considered significant without correction for multiple testing. Since unevenly distributed risk factors for adverse outcomes are possible confounders for the explored hypotheses, univariate logistic regression analyses were performed to identify such risk factors, also including those identified in the unadjusted comparisons of this study and in previous studies. In the following step, multiple logistic regression analyses were carried out, including variables showing significant differences in the univariate analysis in order to identify independent risk factors for NDI, MDI or PDI results < 70 or < 85.

Since multiple regressions done en-bloc for the analysis regarding PDI < 70 revealed non-interpretable results, owing to close similarities between categories, the infection categories as defined above were individually fed into multiple regressions [44]. All analyses were performed using SPSS Statistics 24 (IBM, New York, USA).

## Results

### Study population

**Fig. 1:**
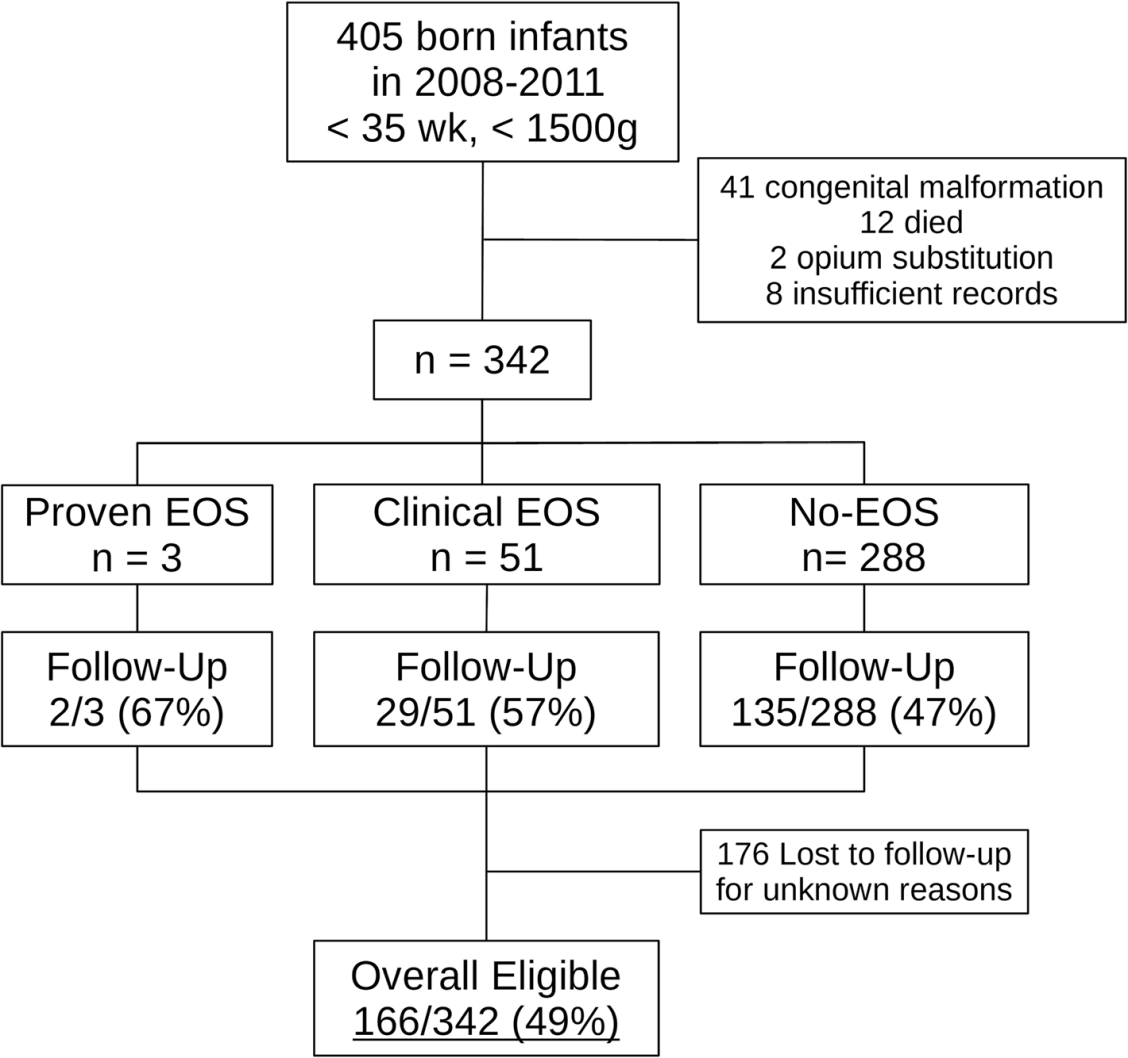
The graph shows the number of patients from enrollment to follow-up

Altogether 405 VLBW infants were born in the study period. Sixty-three were excluded according to the criteria mentioned above, 12 of whom died for reasons not linked to EOS or LOS. For various reasons, including parents moving and/or patients receiving further post-discharge care in other centers closer to their home, 176 patients were lost to follow-up. In the end 166/342 (49%) remained to be analyzed (Fig. 1). Demographic data of those lost to follow-up were compared to patients with follow-up for validation. Baseline demographic characteristics and clinical data of infants with BSIDII- and those without BSIDII-testing were similar. Only the Apgar 10′ score showed a significant difference. However, while being statistically significant, the difference of the average, as well as the median, was less than one score point between both groups (Table 1).

**Table 1:** Demographics

	no Follow-Up	Follow-Up	*p*
*N* = 342 a)	176 (51)	166 (49)	
EOS a)	23 (13)	31 (19)	0.155
Male, a)	81 (46)	69 (42)	0.406
Gestational Age Weeks (wk + d), b)	29 + 4 (± 2 + 3)	29 + 1 (± 2 + 2)	0.085
Birth weight g, b)	1146 (± 282)	1126 (± 278)	0.507
Umbilical artery pH, b)	7.33 (± 0.30)	7.29 (± 0.09)	0.124
Apgar 1′/5′/10′, c)	7 / 8 / 8	7 / 8 / 8	0.250 / 0.144 / 0.022
Apgar 1′/5′/10′ c)	(0; 6–8; 9 / 4; 7–8; 9 / 6; 8–8; 10)	(1; 6–8; 9 / 3; 7–8; 9 / 5; 8–8; 10)	
CRIB-Score, c)	1 (0 / 1–2 / 10)	1 (0 / 1–4 / 12)	0.548
Highest Bilirubin, μmol/l, b)	145.80 (± 28.88)	144.77 (± 33.99)	0.762
Antenatal steroids 1 dose / 2 doses, a)	87 (49) / 53 (30)	90 (54) / 46 (28)	0.478 / 0.624
Ceaseraen delivery, a)	162 (92)	151 (91)	0.589
Patent ductus arteriosus, a)	35 (20)	46 (28)	0.408
BPD, d)	15 (9)	12 (7)	0.693
Retinopathia neonatorum ≥3, d)	0	2 (1)	0.235
Intraventricular haemorrhage ≥3, d)	1 (1)	6 (4)	0.061
Periventricular leukomalacia, d)	0	2 (1)	0.235
Parental Education Score 1/2 d); 3/4 a) ^a^	2 (1) / 10 (6) / 96 (55) / 49 (28)	1 (1) / 9 (5) / 93 (56) / 57 (34)	0.620 / 0.817 / 0.584 / 0.405
Max CrP 72 h postnatal, c)	1.04 (0 / 0–3.91 / 52.37)	1.04 (0 / 0–5.08 / 68.45)	0.748
Leukocytes in first CBC, ×10^9^,b)	9.50 (± 5.86)	9.96 (± 7.04)	0.512
Last Maternal CrP prior to Birth, c)	6.82 (0 / 1.65–17.25 / 118.3)	7.43 (0 / 2.42–21.98 / 70.77)	0.330
PROM < 24 h a) / > 24 h e) /> 1 week, a)	55 (31) / 15 (9) / 29 (16)	39 (23) / 10 (6) / 29 (17)	0.108 / 0.412 / 0.699
Chorioamnionitis, a)	21 (12)	14 (8)	0.374
LOS a)	20 (11)	36 (22)	0.427
Total sepsis symptoms c) ^b^	3 (0 / 1–4 / 7)	3 (0 / 1–4 / 7)	0.127

a) n (%), χ2-test; b) mean, SD, 1-Way ANOVA; c) median, min, IQR, max; Kruskal-Wallis-Test; d) n (%), Fisher’s exact test;

^a^displayed are n of individual parental education score categories as defined in the methods section

^b^displayed is the median total of clinical symptoms associated with EOS as defined in the methods section

*Abbreviations*: *BPD* Bronchopulmonary dysplasia, *CBC* Complete blood count, *CRIB* Clinical risk index for babies, *EOS* Early onset sepsis, *LOS* Late onset sepsis, *PROM* Premature rupture of membranes, *WBC* White blood cell count

In the following step, demographic data of patients with EOS and without EOS were compared. Risk factors for EOS were – not surprisingly – significantly more frequent in the EOS group. Aside from a higher initial CrP, lower gestational age, and a lower birth weight in the EOS-group, infants in this group were more likely to have a higher CRIB-Score, were more prone to develop BPD, and presented with significantly more of the defined clinical symptoms. Mothers of infants with EOS were more likely to have suffered from chorioamnionitis and a premature rupture of membranes (PROM) for longer than a week. Furthermore, a statistically significant difference of the Apgar 5′ score between EOS and no-EOS groups was noted, which was, as in the prior comparison, less than a score point and was thus considered to be clinically irrelevant (Table 2).

**Table 2:** Demographics

	No-EOS	EOS	*p*
*N* = 166	135	31	
Male, a)	54 (40)	15 (48)	0.514
Gestational Age Weeks (wk + d), b)	29 + 2 (± 2 + 2)	27 + 6 (± 2 + 0)	0.001
Birth weight g, b)	1160.56 (± 267.62)	974.97 (± 276.40)	0.001
Umbilical artery pH, b)	7.30 (± 0.09)	7.29 (± 0.08)	0.811
Apgar 1′/5′/10′, c)	7 / 8 / 8	7 / 8 / 8	0.125 / 0.041 / 0.057
Apgar 1′/5′/10′ c)	(1; 6–8; 9 / 3; 7–8; 9 / 5; 8–9 10)	(4; 6–8; 8 / 3; 7–8; 9 / 3; 8–8; 9)	
CRIB-Score, c)	1 (0 / 0–3 / 11)	3 (0 / 1.75–5.25 / 12)	0.001
Highest Bilirubin, μmol/l, b)	143.85 (± 23.44)	148.74 (± 62.31)	0.472
Antenatal steroids 1 dose / 2 doses, a)	72 (53) / 35 (26)	18 (58) / 11 (35)	0.633 / 0.284
Ceaseraen delivery, a)	121 (90)	30 (97)	0.366
Patent ductus arteriosus, d)	36 (27)	10 (32)	0.830
BPD, f)	6 (4)	6 (19)	0.011
Retinopathia neonatorum ≥3, d)	2 (1)	0	0.999
Intraventricular haemorrhage ≥3, d)	4 (3)	2 (6)	0.312
Periventricular leukomalacia, d)	1 (1)	1 (3)	0.340
Parental Education Score 1/2 d); 3/4 a); ^a^)	1 (1) / 7 (5) / 76 (56) / 47 (35)	0 / 2 (6) / 17 (55) / 10 (32)	0.999 / 0.667 / 0.999 / 0.999
Max CrP 72 h postnatal, c)	0.62 (0 / 0–1.525 / 8.37)	14.36 (5.37 / 9.5–28.07 / 68.45)	0.000
Leukocytes in first CBC, ×10^9^, b)	9.9 (±5.71)	10.22 (±11.09)	0.824
Leukopenia a)	9 (7)	10 (32)	0.000
Leukocytosis d)	4 (3)	3 (10)	0.122
Combinded abnormal WBC a)	13 (10)	13 (42)	0.000
Thrombocytes in first CBC, × 10^9^, b)	181.8 (±69.7)	173.4 (±71.7)	0.547
Thrombocytopenia, a)	40 (30	14 (45)	0.146
Severe thrombocytopenia, d)	3 (2)	2 (6)	0.234
Last Maternal CrP prior to Birth,c)	7.21 (0 / 2.49–20.41 / 70.77)	8.8 (0 / 2.04–29.16 / 64.93)	0.541
PROM < 24 h / > 24 h /> 1 week, d)	36 (27) / 10 (7) / 17 (13)	3 (10) / 0 / 12 (39)	0.059 / 0.211 / 0.001
Chorioamnionitis, d)	8 (6)	6 (19)	0.026
LOS d)	27 (20)	9 (29)	0.211
Age at BSIDII, month, e)	24 (23–30)	25 (24–31)	0.323
Total sepsis symptoms c) ^b^	3 (0 / 2–3.5 / 6)	4 (2 / 3–5 / 7)	0.000

a) n (%), χ2-test; b) mean, SD, 1-Way ANOVA; c) median, min, IQR, max; Kruskal-Wallis-Test; d) n (%), Fisher’s exact test; e) median, min – max, Mann-Whitney-U-Test

^a^displayed are n of individual parental Education score categories as defined in the methods section

^b^displayed is the median total of clinical symptoms associated with EOS as defined in the methods section

*Abbreviations*: *BPD* Bronchopulmonary dysplasia, *BSID* Bayley Scales of Infant Development II, *CBC* Complete blood count, *CRIB* Clinical risk index for babies, *EOS* Early onset sepsis, *LOS* Late onset sepsis, *PROM* Premature rupture of membranes, *WBC* White blood cell count

Of the analyzed 166 infants, two had culture-proven sepsis (one caused by a gram-negative pathogen, one by a fungus), 29 had clinical sepsis, and none had NEC (Fig. 1). Of the 29 cases of clinical EOS, five had increased IL-6 serum levels and 13 had abnormal WBCs. Increase in IL-6 and abnormal WBC only coincided in one case. Five patients with clinical sepsis had been exposed to prenatal maternal antibiotics. Thirty-six patients evenly spread among the study groups developed LOS, one even developed meningitis during the hospital stay but had not had EOS before (Table 2). One hundred thirty-five patients did not meet the criteria of EOS.

### Outcome assessment

**Fig. 2:**
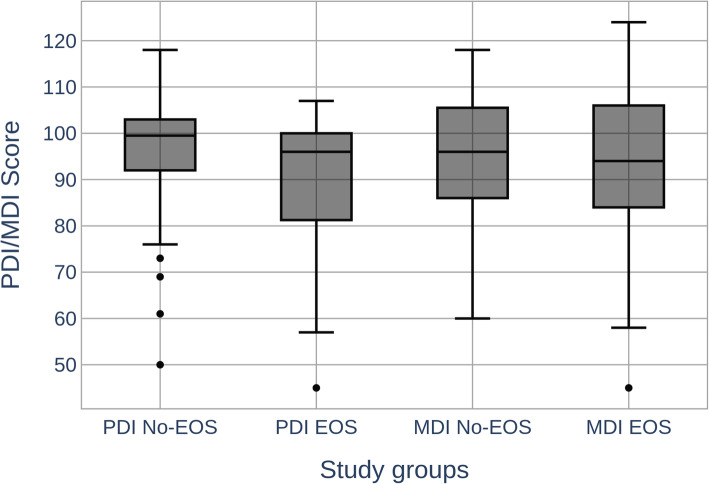
Displayed are PDI and MDI scores for infants in the EOS and no-EOS groups. Presented are median, quartiles, lower fence and maxima. For MDI Scores in the No-EOS group a minimum is presented. PDI, median, IQR: EOS 96 (80 – 106) No-EOS 99,5 (92 – 103) p = 0,029*. MDI: EOS 96 (86 – 106) No-EOS 94 (84 – 106) *p* = 0,771*. *Student‘s T-Test/Welch-Correction

BSIDII examinations were carried out at a median of 24 months (Range 23 – 31 months) corrected age with no significant difference between the study groups (Table 2). Results of these follow-up examinations are displayed in Table 3 and Fig. 2. MDI values were similar in both study groups, however PDI values were significantly lower in the EOS group. In particular, PDI values < 70 showed a significant association with infection while PDI values < 85 only showed a weak association. Moreover, the occurrence of cerebral palsy was significantly higher in the EOS group. Altogether, NDI as defined above was significantly more frequent in the EOS group (Table 3). Two infants of the EOS-group had extremely low MDI or PDI values (Fig. 2). In the case of one bilingual patient, a MDI of 45 was measured due to poor concentration on the test items. Another infant with a PDI of 45 had been born extremely early and with a severe intrauterine growth retardation (GA 25 + 6 weeks, birth weight 445 g).

**Table 3:** Outcome

	EOS *n* = 31	No-EOS = 135	*p*	OR	95% CI	*p*
NDI, a)	7 (23,58%)	11 (8,15%)	0.015	3.288	1158 – 9336	0.025
PDI < 70, a)	6 (19,35%)	3 (1,65%)	0.002	10.56	2476 – 45,035	0.001
MDI < 70, a)	3 (9,68%)	6 (4,44%)	0.371	2.304	0,543 – 9771	0.318
PDI < 85, b)	8 (25,81%)	18 (13,33%)	0.088	2.261	0,879 – 5818	0.091
MDI < 85, b)	7 (23,58%)	37 (27,41%)	0.583	0.773	0,307 – 1944	0.584
Cerebral Palsy, a)	4 (12,09%)	3 (1,65%)	0.014	6.519	1379 – 30,809	0.018

a) n, %, Fisher‘s Exact Test; b) n, %, χ2-test

*Abbreviations*: *MDI* Motor Development Index, *PDI* Psychomotor Developmental Index, *NDI* Neurodevelopmental impairment

Logistic regression models showed a few significant risk factors for low MDI or PDI scores and NDI (Tables 4, 5, 6). In the multiple logistic regression analysis, MDI scores < 70 showed a significant association with the CRIB-Score. Risk factors for PDI < 70 (in the different analyses) were chorioamnionitis and a high CRIB-Score. No significant individual risk factors for the combined outcome NDI were identified in the multiple logistic regression model. As secondary outcomes MDI scores < 85 showed a weak association with BPD and a significant risk factor for PDI < 85 was IVH (Table 7).

**Table 4:** Regression analysis PDI < 70

	Univariable Model	*p*	Multivariable Model a)	*p*	Multivariable Model b)	*p*	Multivariable Model c)	*p*
	OR (95% CI)		OR (95% CI)		OR (95% CI)		OR (95% CI)	
EOS	10.480 (2.457 – 44.696)	0.001	5.546 (0.568 – 54.138)	0.141				
Proven EOS	19.375 (1.108 – 338.807)	0.042			14.618 (0.179 – 1193.351)	0.232		
Clinical EOS	6.550 (1.644 – 26.104)	0.008					2.701 (0.306 – 23.848)	0.371
PROM > 1 week	4.192 (1.052 – 16.707)	0.042	4.176 (0.641 – 27.201)	0.135	2.811 (0.407 – 19.390)	0.294	4.572 (0.681 – 30.695)	0.118
Chorioamnionitis	6.591 (1.448 – 29.997)	0.015	6.291 (0.839 – 47.175)	0.074	7.292 (1.029 – 51.677)	0.047	8.724 (1.314 – 57.919)	0.025
Max CrP 72 h postnatal	1.048 (1.009 – 1.089)	0.016	1.006 (0.902 – 1.099)	0.902	1.050 (0.982 – 1.123)	0.153	1.022 (0.937 – 1.116)	0.618
CRIB-Score	1.276 (1.042 – 1.564)	0.019	1.311 (0.968 – 1.773)	0.080	1.345 (1.015 – 1.782)	0.039	1.345 (1.000 – 1.808)	0.050
Highest Bilirubin	1.014 (1.000 – 1.028)	0.044	1.002 (0.981 – 1.023)	0.860	0.998 (0.978 – 1.018)	0.815	0.999 (0.979 – 1.020)	0.952
BPD	4.171 (0.764 – 22.767)	0.099						
Periventricular leukomalacia	19.375 (1.108 – 338.807)	0.042	7.159 (0.246 – 207.978)	0.252	12.907 (0.485 – 343.342)	0.127	7.735 (0.277 – 215.872)	0.228

Displayed are parameters showing a weak association (*p* < 0,1). Included in the multiple regression models were variables with *p* < 0,05 and a) with the combined EOS variables, b) proven EOS and c) only clinical EOS

*Abbreviations*: *CRIB* Clinical risk index for babies, *EOS* Early Onset Infection, *PROM* Premature rupture of membranes

**Table 5:** Regression analysis MDI < 70

	Univariable Model	*p*	Multivariable Model *p* < 0,05	*p*
	OR (95% CI)		OR (95% CI)	
Proven EOS	19.500 (1.115 – 340.984)	0.042	25.559 (1.291 – 506.152)	0.033
CRIB-Score	1.246 (1.026 – 1.514)	0.027	1.269 (1.038 – 1.552)	0.020
BPD	4.200 (0.770 – 22.922)	0.097		

Displayed are parameters showing a weak association (*p* < 0,1). Included in the multiple regression models were variables with *p* < 0,05

*Abbreviations*: *BPD* Bronchopulmonary dysplasia, *CRIB* Clinical risk index for babies, *EOS* Early Onset Infection

**Table 6:** Regression Analysis NDI

	Univariable Model	*p*	Multivariable Model *p* < 0,05	*p*
	OR (95% CI)		OR (95% CI)	
EOS	3.29 (1.16 – 9.34)	0.025	2.652 (0.804 – 8.743)	0.109
Clinical EOS	2.58 (0.88 – 7.554)	0.083		
CRIB-Score	1.22 (1.04 – 1.42)	0.012	1.121 (0.928 – 1.353)	0.236
Highest Bilirubin	1.01 (1.00 – 1.03)	0.046	1.007 (0.992 – 1.022)	0.377
Intraventricular haemorrhage ≥3	9.67 (1.79 – 52.19)	0.008	4.309 (0.535 – 34.726)	0.170

Displayed are parameters showing a weak association (*p* < 0,1). Included in the multiple regression models were variables with *p* < 0,05

*Abbreviations*: *CRIB* Clinical risk index for babies, *EOS* Early Onset Infection

**Table 7:** Secondary Outcome

	Univariable Model	*p*	Multivariable Model *p* < 0,05	*p*
	OR (95% CI)		OR (95% CI)	
Regression analysis MDI < 85				
Gestational Age	0.973 (0.951 – 0.996)	0.022	0.994 (0.960 – 1.030)	0.756
Birthweight	0.998 (0.997 – 0.999)	0.002	0.999 (0.997 – 1.002)	0.578
Male Gender	2.023 (0.966 – 4.237)	0.062		
CRIB-Score	1.282 (1.124 – 1.462)	0.000	1.145 (0.930 – 1.411)	0.202
BPD	6.556 (1.865 – 23.040)	0.003	3.392 (0.853 – 13.485)	0.083
Regression analysis PDI < 85				
EOS	2.242 (0..871 – 5769)	0.094		
PROM > 1 week	2.497 (0.962 – 6.480)	0.060		
Gestational Age	0.955 (0.926 – 0.984)	0.003	0.983 (0.938 – 1.031)	0.480
Birth weight	0.997 (0.996 – 0.999)	0.002	1.00 (0.997 – 1.003)	0.952
Apgar 10‘	0.548 (0.305 – 0.986)	0.045	0.647 (0.289 – 1.45)	0.290
CRIB-Score	1.352 (1.168 – 1.564)	0.000	1.128 (0.863 – 1.475)	0.377
BPD	6.650 (1.953 – 22.645)	0.002	3.408 (0.775 – 14.994)	0.105
Intraventricular haemorrhage ≥3	32.857 (3.657 – 295.224)	0.002	18.137 (1.676 – 196.218)	0.017
LOS	3.939 (0.798 – 19.453)	0.092		

Displayed are parameters showing a weak association (*p* < 0,1). Included in the multiple regression models were variables with *p* < 0,05

*Abbreviations*: *BPD* Bronchopulmonary dysplasia, *CRIB* Clinical risk index for babies, *EOS* Early Onset Infection, PROM premature rupture of membranes

## Discussion

Infections remain a threat for VLBW-infants, as they are still associated with short- and long-term sequelae and an increased risk of death [2–4, 11]⁠.

Our findings support the hypothesis that VLBW-infants with EOS in our study cohort had an increased risk for poor neurodevelopmental outcomes at 2 years of age. In fact, the risk was three times higher than in the control group without EOS. It appears that the psychomotoric development was more affected than the mental development. PDI scores were significantly lower in infants who had EOS, and there was an increased number of infants who had cerebral palsy in the EOS group. The differences were not explained by differences in other confounding variables according to the multiple logistic regression results.

Looking at PDI scores, chorioamnionitis as a known maternal precursor for EOS showed a significant negative impact [45]. IVH as an individual affliction of the neonate showed a significant negative impact on the PDI as well. Low MDI scores were weakly associated with BPD in our models. A higher CRIB-Score was related to poorer outcomes in both MDI and PDI.

Our findings concur with previous studies. Fairly recently Ferreira et al. [16]⁠ identified clinical sepsis in a Brazilian population as an individual predictor of poorer neurodevelopmental outcomes. In particular the psychomotoric development seemed to be more affected, as well. Research done by Schlapbach et al. [8] showed proven sepsis to be an individual predictor of poorer neurodevelopmental outcomes in infants with EOS and LOS. Before that study, cohorts of Stoll et al. [6] and Bassler et al. [20] had yielded similar results. An association of NDI with culture-proven EOS was shown very recently by Mukhopadhyay et al. [11]⁠ on a cohort of extremely preterm infants (gestational age < 27 weeks) born between 2006 and 2014. Sepsis suspected solely on clinical symptoms, however, did not have a significant association.

As demonstrated here, maternal chorioamnionitis was also shown to affect the PDI in neonates by Klinger et al. [21]. Furthermore, neonatal sepsis has also been established as a risk factor for cerebral palsy [6, 8, 46].

When comparing our odds ratios to previous studies, the overall risk profile regarding neurodevelopmental impairment caused by EOS seems to have remained fairly similar since the 1990s.

The mechanisms as to how neonatal sepsis causes brain injury, however, notably without direct CNS involvement such as intracranial hemorrhage or meningitis, are still unknown. A study using MRI imaging has shown white matter injury in patients with recurrent postnatal infections [47]. In general a higher vulnerability to injury because of the immaturity of the developing brain is postulated [7, 9]. Several causative mechanisms have been suggested, including cytokines being transported actively across the intact blood brain barrier, a potential mechanism through the activation of the hypothalamic-pituitary-adrenal-axis, a pathway across the blood brain barrier at circumventricular organs, and a ‘leak’ across an intact blood brain barrier with altered permeability through inflammation or cytokines being produced by cells infiltrating the CNS [5, 7]. Although it appears that the inflammatory cytokine response precedes and contributes to brain damage, there are additional risks of brain damage during EOS through haemodynamic instability and respiratory disease as well. Hypoxaemia and pathological alterations in cerebral blood flow may pose a risk for neurodevelopmental outcome by themselves [6, 7, 20]. BPD as an individual risk factor of NDI is associated with cytokine release from the lungs, sepsis, and with an increased exposure to ventilation and oxygen in infants with EOS [21].

Avoiding EOS continues to be challenging in the prevention of NDI. Even optimized therapy protocols seem to have resulted, if at all, in only a minor reduction in NDI in affected patients. Reducing EOS itself may be most effective in increasing the proportion of infants surviving without NDI [48]. Identifying EOS remains a major obstacle in the prevention of NDI, with the gold standard still being proof by culture. A study by Mikhael et al. [22]⁠ of 1593 neonates ≥ 23 weeks’ gestation, who underwent an EOS evaluation ≤ 72 h postnatal due to clinical signs and an elevated risk for EOS, found only 9 cases (0.56%) of proven EOS. The potentially large difference between incidence of culture proven EOS, and patients presenting with clinical symptoms of an infection, was demonstrated in other studies as well, most recently by Mukhopady et al. [8, 11, 16]. In their study of the impact of EOS and antibiotic use on patients with an extremely low birth weight (< 1000 g) culture-confirmed EOS occurred in 2.3% of the patients, even though 48.6% of them received antibiotic treatment for more than 5 days.

Similar differences between incidence rates of clinically diagnosed sepsis and culture proven sepsis can be seen in the German ‘Neonatalerhebung’ (a registry of neonatal births in all German perinatal centers), as well as in studies carried out in developing countries [10, 16, 49]⁠. A discussion as to how to define sepsis in different pediatric patient collectives was led in the past years [14, 50]⁠. Without a universally accepted sepsis definition, especially for neonatal patients, researchers have made attempts similar to ours using clinical and paraclinical parameters to define clinical EOS in their studies [6, 8, 11]⁠. Fairly recently, following the Sepsis-3 consensus definition in adults, promising steps have been made to enter definitions of organ failure using scoring systems to improve identification of sepsis [14, 51–53]⁠. However, the wide range of pediatric patients, and even of neonates, remains an obstacle to these efforts. Wynn et al. [53]⁠ introduced a neonatal sequential organ failure score (nSOFA) including a risk calculator for LOS patients. They aim to validate and possibly adapt the nSOFA for EOS patients in future multi-center research, too. In fact, recent research of the group has shown very promising results validating the nSOFA for LOS patients [54]⁠.

Furthermore, a priori risk stratification scores have been established, which have lead to the Kaiser EOS calculator, using maternal as well as patients’ characteristics (e.g. maternal GBS status, PROM, gestational age and clinical status) to allow an identification of patients at a higher risk of EOS [4, 12, 19, 26]⁠. With a more accurate estimation of neonates at risk, clinicians are able to monitor high risk patients more closely, and reduce the number of patients being treated unnecessarily with antibiotics, thus reducing the risks of antibiotic treatment itself [4, 26, 55]⁠. This risk reduction is directly linked to short and long term neonatal outcomes such as, among others, increased risk of NEC, fungal infections, and death, or alterations to the microbiome, allergies, and autoimmune diseases [4, 55]⁠. In fact, Mukhopadhyay et al. [11]⁠ reported that antibiotic treatment for 5 days or more, in the absence of culture proven sepsis, by itself showed a trend towards adverse neurodevelopmental outcome, which might be explained by a portion of these infants actually having sepsis, but negative cultures. Highly sensitive and specific blood biomarkers for sepsis were unfortunately not considered.

A few limitations of this study need to be addressed. The single center design and the focus on EOS of our study avoided inter-center variations, but limited the available sample size. Only two cases of culture-proven EOS were available in the study group. However, with known incidence rates and research into the sensitivity of blood culture in neonatal care, our reported numbers fall into the expected range [12, 22]⁠. Additionally, in five cases prenatal maternal antibiotics may have caused sterile blood cultures in clinical EOS patients. Some potentially confounding conditions, which have shown an impact on NDI in previous studies, (i.e. ROP, NEC, IVH), were too rare in our cohort to be statistically analyzed [1, 6, 8]. Owing to the retrospective design, not all important parameters were measured in all infants, and some, such as IL-6, were only sparsely measured at all. Other findings associated with EOS, such as severe thrombocytopenia, were too rare to show significant differences between the study groups. This was to be expected due to, first of all, a wide range of morbidities causing early onset thrombocytopenia, and, second of all, as shown in prior research, only a small increase in the odds of EOS with low platelet counts [56]⁠. As discussed above, the maternal GBS status would have further improved the stratification of our study groups, but was unfortunately not accessible to us, since prenatal GBS cultures are not part of standard care in Germany [19]⁠. The surrounding sociogeographic structures of our perinatal center led to a proportion of patients being lost to follow-up. Especially in the first year of our study, there was a high proportion lost to follow-up as follow-up exams only became mandatory in Germany in 2009. The rates of infection and basic demographic data were very similar in both groups with and without follow-up, indicating that our study group is representative (Table 1). Demographic differences in the comparison between the EOS and No-EOS groups can be explained on the one hand by known risk factors of EOS, such as prematurity, low-birth weight or chorioamnionitis, and on the other by the differentiation of the group itself, i.e. CrP and WBC values. The difference in CRIB-scores may be explained by the overlap of criteria included in the score with the above mentioned parameters. Such differences were to be expected [9]⁠.

The large extent of clinical and paraclinical factors linked to NDI in premature infants by themselves may impede determining the individual cause of an adverse outcome, especially as such factors may be linked to EOS as well. In our study such parameters (among others lower birth weight, lower gestational age, BPD, antibiotic treatment) showed significant differences between the study groups. In fact, the CRIB score was, not surprisingly, identified as a risk factor in our regression analyses, since it employs a combination of the above mentioned parameters. Additionally physiologic clinical alterations in the neonatal period may be mistaken for pathological sepsis symptoms. Therefore studies into EOS and LOS using clinical definitions need to be interpreted with caution, since clinical presentations may have a low specificity. However, as Stoll et al. [12]⁠ recently reported, in their study cohort nearly all infected infants had signs of instability within 72 h after birth and the clinical diagnosis remains essential in the early detection of infection [19, 26, 27]⁠. We aimed to overcome these weaknesses through a combination of clinical and paraclinical findings, as was shown in prior research to improve specificity [4, 13, 15, 19, 23, 24, 26]⁠. The correlation between significant differences in EOS and no-EOS groups in the occurrence of clinical symptoms and blood biomarkers, as well as the consistency of our findings with prior research, could be interpreted in favor of our clinical EOS definition and thus pointing to EOS as cause of NDI (Table 2).

The differences in Apgar scores between demographic groups of less than one score point have no predictive value for neurodevelopmental outcomes, making a bias unlikely [57] (Tables 1 and 2). The limited sample size and low numbers in specific adverse outcomes limited the ability to perform multivariate analyses, as seen to some extent in previous studies as well [8, 16]⁠. Therefore, odds ratios are to be interpreted with caution as, here, they tended to have rather wide confidence intervals [44]. In addition, like many other studies evaluating neurodevelopmental outcomes, our study is based on outcomes determined in a single assessment at the corrected age of 2 years [58]⁠. Later cognitive and neuromotor outcomes, such as specific learning difficulties or milder motor dysfunctions in light of our tendency for poorer PDI outcomes, are not part of the study because such data is not available [8, 59, 60]. Another limitation to our study, as well as others, is death after discharge from hospital not being recorded as a competing outcome [6, 8, 16]⁠. However, most deaths in EOS patients occur in the postnatal period in hospital and no deaths linked to EOS were recorded in our patients [12]⁠.

Strengths of our study include a focused assessment into the effects of EOS, and an EOS definition with improved sensitivity and specificity by using established blood infection markers. LOS, as mentioned above, was distributed evenly among the study groups, making a bias herein unlikely. Since this is a single-center study, our study cohort was assessed by the same definitions and treated according to the same standard operating procedures. The BSID-II exam was carried out by a single experienced neonatologist (AB).

## Conclusion

A significant association of EOS with an increased risk of poor neurodevelopmental outcomes in VLBW-infants was demonstrated. Measures for earlier detection, prevention, and improved treatment standards have shown a reduction in infections, while the risk of neurodevelopmental impairment in patients suffering from EOS seems to be fairly constant. Hence, while there has been improvement, neurodevelopmental impairment caused by EOS and the pathogenesis need to stay in focus in future research to further improve understanding and, consequently, therapy. Perinatal standard operating procedures for mother and child need to be focused on prevention, and earlier detection for a quick therapeutic reaction.

## Data Availability

The datasets used and/or analyzed during the current study are available from the corresponding author upon reasonable request. (Correspondence to tjark.ortgies@gmx.de).

## Abbreviations

BPD: Bronchopulmonary dysplasia
BSIDII: Bayley Scale of Infant Development II
CBC: Complete blood count
CNS: Central nervous system
CRIB-Score: Clinical Risk Index for Babies
EOS: Early-onset-sepsis
GBS: Group-B-Streptococcus
GMFCS: Gross Motor Function Classification System
IL-6: Interleukin-6
IVH: Intraventricular hemorrhage
LOS: Late-onset-sepsis
MDI: Mental Developmental Index
NDI: Neurodevelopmental impairment
NEC: Necrotizing enterocolitis
NICU: Neonatal intensive care unit
PDA: Patent ductus arteriosus
PDI: Psychomotor Developmental Index
PROM: Premature rupture of membranes
ROP: Retinopathy of prematurity
SIRS: Systemic inflammatory response syndrome
VLBW: Very low birth weight (< 1500 g)
WBC: White blood cell count
